# Effects of Traditional Flood Irrigation on Invertebrates in Lowland Meadows

**DOI:** 10.1371/journal.pone.0110854

**Published:** 2014-10-23

**Authors:** Jens Schirmel, Martin Alt, Isabell Rudolph, Martin H. Entling

**Affiliations:** Institute of Environmental Science, University of Koblenz-Landau, Landau, Germany; University of Waikato (National Institute of Water and Atmospheric Research), New Zealand

## Abstract

Lowland meadow irrigation used to be widespread in Central Europe, but has largely been abandoned during the 20^th^ century. As a result of agri-environment schemes and nature conservation efforts, meadow irrigation is now being re-established in some European regions. In the absence of natural flood events, irrigation is expected to favour fauna typical of lowland wet meadows. We analysed the effects of traditional flood irrigation on diversity, densities and species composition of three invertebrate indicator taxa in lowland meadows in Germany. Unexpectedly, alpha diversity (species richness and Simpson diversity) and beta diversity (multivariate homogeneity of group dispersions) of orthopterans, carabids, and spiders were not significantly different between irrigated and non-irrigated meadows. However, spider densities were significantly higher in irrigated meadows. Furthermore, irrigation and elevated humidity affected species composition and shifted assemblages towards moisture-dependent species. The number of species of conservation concern, however, did not differ between irrigated and non-irrigated meadows. More variable and intensive (higher duration and/or frequency) flooding regimes might provide stronger conservation benefits, additional species and enhance habitat heterogeneity on a landscape scale.

## Introduction

Semi-natural grasslands are key habitats for biodiversity conservation and an integral part of the Central European cultural landscape [1]–[3]. They are among the most species-rich habitats and serve as refuges for several rare and endangered species [2]–[4]. Regular disturbance due to traditional management permits the coexistence of numerous species in semi-natural grasslands [2]. During the last decades however, semi-natural grassland have dramatically declined in Central Europe and further declines to less than 50% of the current area are predicted [5], [6]. Major causes are agricultural intensification and the abandonment of traditional management. The latter is mainly due to the reduced cost-effectiveness of traditional land use practices [5], [7]. Agricultural intensification practices for semi-natural meadows include higher fertilizer and herbicide applications, earlier and more cuts per year, and the use of modern mowing techniques [8]. This results in eutrophic, structurally poor, and homogeneous meadows with negative impacts on diversity, species composition, and ecosystem processes [9].

Until the early 20^th^ century, meadow irrigation was widespread in Central Europe to increase hay yield [10]. For example, around 1900 in some regions of Germany, irrigated meadows made up about 60% of the total grassland [11]. The main effects of irrigation were nutrient input, topsoil humidification, and extension of the vegetation period. Nowadays irrigation practices are mostly abandoned and traditionally irrigated meadows with their associated species are restricted to few remnant areas [12]. Thanks to agri-environment schemes (e.g. in form of compensation payments), nature conservation efforts, and due to mitigation and compensation measures, the traditional irrigation practices could be maintained or re-established in some European regions [13]. However, the value of agri-environment schemes is under debate and further analyses of management strategies are necessary [14], [15]. Therefore, it is of growing interest to determine, how traditional irrigation practices affect biological diversity. In this context, Riedener et al. [16] recently showed that changes in irrigation techniques have influenced some aspects of plant and gastropod diversity in Swiss mountain hay meadows. However, knowledge of the influence of traditional meadow irrigation on invertebrate diversity and composition is still poorly understood, and this is especially true for flood irrigation in lowland regions.

The objective of this study was to analyse whether traditional flood irrigation in lowland meadows has an effect on invertebrate diversity and species composition. Irrigation is assumed to create small-scale differences in moisture and sediment conditions which may increase microhabitat and vegetation heterogeneity [16]. In accordance to the habitat-heterogeneity-hypothesis irrigation might therefore have positive effects on local species richness [17]–[20]. Moreover, flood irrigation in our study area is conducted in a similar way among irrigated sites, but differs in timing and intensity. This may lead to non-uniform moisture conditions among irrigated meadows with heterogeneous species compositions and higher beta diversity. To investigate these predictions we conducted a field survey in the ‘Queichtal’ in Rhineland-Palatine, Germany. We compared traditionally flood-irrigated meadows with meadows, where there has been no irrigation for at least thirty years. We focused on orthopterans, carabids and spiders, which are found at different trophic levels within grassland food-webs and occur in different vegetation layers. Orthopterans (Orthoptera) are mostly grass-dwelling herbivores, where they are often both the main invertebrate consumers and the main food source [21]. Most carabids (Coleoptera: Carabidae) are ground-dwelling predators, but some are scavengers and herbivores [22]. Spiders (Araneae) inhabit both the ground and field layer, often in high abundances, and are predatory [23]. All three arthropod groups have been used as indicators of ecosystem conditions and habitat quality (orthopterans: [24], [25]; carabids: [26], [27]; spiders: [28], [29]).

We addressed the following hypotheses: (i) Flood irrigation increases the local diversity of orthopterans, carabids, and spiders compared to non-irrigated lowland meadows. (ii) Flood irrigation leads to higher beta diversity relative to non-irrigated meadows. (iii) Flood irrigation shifts species assemblages towards more moisture-dependent species and those of higher conservation concern than species in non-irrigated meadows. Based on our findings we discuss if traditional flood irrigation can be useful for conserving biodiversity of semi-natural grassland species.

## Materials and Methods

### Ethics statement

Invertebrates were collected with the permission 42/553-254 from the Struktur- und Genehmigungsdirektion Süd (federal state authority of Rhineland-Palatine, Germany). Additionally, we obtained permissions from all private farmers and landowners to conduct the field work on their meadows.

### Study sites

**Figure 1:**
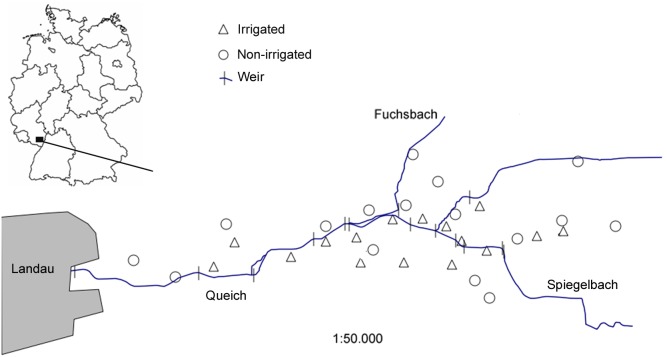
Position of the study area ‘Queichtal’ in Germany (small figure) and of the 32 study sites.

The study was conducted in 2012 in the ‘Queichtal’ in the Upper Rhine valley in Rhineland-Palatine, Germany (Fig. 1). With a length of ∼51 km, the river Queich is an important drainage system of the adjacent low mountain range ‘Pfälzerwald’ into the Rhine. Soils of the alluvial sediments are sandy to loamy [30]. Annual mean temperature in this region is 10.5°C (station Neustadt) and annual mean precipitation is 667 mm (station Landau; German Weather Service). The studied section of the Queichtal covers about 700 ha, is part of the NATURA 2000 network, and is thus protected by the EU habitats Directive [31].

Due to the predominance of moist soils with low cation availability, land use in the Queichtal is dominated by forest and grassland with different management and irrigation regimes. The formerly widespread traditional flood irrigation of lowland meadows was almost totally abandoned after the Second World War and is nowadays only applied in a few remnant areas. For flood irrigation the water of the river Queich (or the tributaries ‘Spiegelbach’ and ‘Fuchsbach’) is dammed by weirs (Fig. 1) and delivered to the meadows by open ditches where it slowly flows over the ground (‘lowland irrigation type’; [10]). Meadows are irrigated on average four times per year between April and August and each irrigation event lasts for 1–3 days.

A total of 32 meadows were selected stratified to meadow irrigation practice (yes or no) and fertilization (yes or no) (Table S1). Half of the meadows were traditionally irrigated and days of irrigation ranged from 4 to 12 days per year. On the other 16 meadows irrigation ceased more than 30 years ago. Half of the irrigated and not irrigated meadows were fertilized (with a maximum of ∼60 kg N•ha-1•yr-1). Meadows were normally mown twice per year and extensive winter grazing by sheep occured on all meadows.

On each meadow we selected a 50×50 m plot with a minimum distance of 100 m from the nearest plot and 10 m to the next ditch to minimise edge effects. Irrigated and non-irrigated meadows did not differ significantly in mean distance to the nearest forest (t-test: t_30_ = 0.563, P = 0.578) and to the nearest permanent water (t-test: t_30_ = 0.529, P = 0.601). Permanent water was defined as any standing and flowing water body which permanently contained water.

### Management and environmental parameters

Land use data on irrigation practice (yes or no) and fertilization (yes or no) were collected through on-site observations and interviews with landowners and local farmers. Plant species were recorded in three randomly selected 3×3 m subplots per plot in May/June before the first cut (unpublished data). For subsequent analyses, data of the three subplots were averaged and we calculated the unweighted mean Ellenberger indicator values for moisture (in the following ‘humidity’ to avoid confusion with animal species moisture indicator values) and nitrogen for a description of local habitat conditions. As species can be influenced by patch isolation [32], we calculated the distance (m) to permanent water and to forest for each sampling location in Google Earth [33].

### Invertebrate sampling

Orthopterans were sampled once per plot during their main activity period in August with a box-quadrat. The box-quadrat is a very effective method for sampling orthopteran densities [34]. The box-quadrat we used had an area of 2 m^2^ (1.41×1.41 m) with white gauze-covered sides of 0.8 m height and was randomly dropped at 20 different locations per plot (total sampled area = 40 m^2^ per plot). Collected individuals were determined to species level directly in the field using Bellmann [35] and then released.

Carabids and spiders were sampled using pitfall traps (6.5 cm in diameter, 7 cm deep) filled to one third with a 50% propylene-glycol solution. Per plot, four pitfall traps (N = 160 traps) were randomly installed with a minimum distance of 5 m to each other. Traps were exposed for two sampling periods from 03 to 24 April and again from 12 to 28 June. Carabids and spiders were determined to species level using the identification keys of Müller-Motzfeld [36] (carabids) and Roberts [37] (spiders). The four traps per plot were treated as a unit and data from both sampling periods were combined to obtain one dataset for further analyses. Due to loss and damage of some pitfall traps, we finally included 28 plots (N = 14 per meadow type each with N = 7 fertilized) in the data analyses of carabids and spiders.

### Data analysis

Species were classified as species of conservation concern when they were listed in regional red lists (all species belonging to the categories ‘1’, ‘2’, ‘3’, ‘4’, and ‘V’; orthopterans: [38]; carabids: [39]; spiders: [40]). For species moisture dependence we used published moisture indicator values. For orthopterans, transformed moisture values were obtained from Maas et al. [41] (Table S2). The values range from ‘1’ (strongly xerophilic) to ‘5’ (strongly hygrophilic). For carabids, moisture values range from ‘0’ (most xerophilic) to ‘9’ (most hygrophilic) according to Irmler and Gürlich [42]. For spiders, we used the moisture values of Entling et al. [43]. For a better comparison to the other taxa we transformed values by 1−x, i.e. species with the lowest value ‘0’ are most xerophilic and species with the highest value ‘1’ are most hygrophilic. For each species we calculated the spearman rank correlation coefficient between species density and irrigation to express their ‘species irrigation affinity’ for our study area. Relationships between species irrigation affinities and species moisture indicator values (based literature data) were analysed using linear models.

The effect of irrigation on species richness and densities of orthopterans, carabids, and spiders were analysed using Poisson GLM’s for count data. Similarly, the irrigation effect on the combined number of species of conservation concern of all taxa (N = 28 sites) was analysed. In cases of overdispersion, we corrected the standard errors using a quasi-GLM model [44]. Differences in Simpson diversity (1−D) between irrigated and non-irrigated meadows were tested with non-parametric Wilcoxon rank sum test, because assumptions for a t-test were violated.

Community differentiation (beta diversity) among irrigated and non-irrigated meadows was analysed using the homogeneity of multivariate dispersions based on the Sørensen similarity of species presence-absence data (using the command ‘betadisper’ in the R package ‘vegan’) [45]. For each taxon, an ANOVA was used to test for differences between the multivariate dispersions of both meadow types.

Effects of the management and environmental variables on species composition of orthopterans, carabids, and spiders were analysed with a permutational multivariate ANOVA (command ‘adonis’ in R package ‘vegan’; [46]). Predictor variables were the two factors irrigation (yes or no) and fertilization (yes or no), the two local habitat parameters humidity and nitrogen (mean Ellenberger indicator values), and the two landscape parameters distance to permanent water and distance to forest. As a distance measure the Bray-Curtis distance was used. Significance of environmental variables was tested with permutation tests (999 permutations) with pseudo-F ratios. Variation of species compositions were visualised using nonmetric multidimensional scaling (NMDS) with the command ‘metaMDS’ in R package ‘vegan’. Again, the Bray-Curtis distance was used as a distance measure. All statistical analyses were done in R 2.12.2 [47].

## Results

### General results

**Figure 2:**
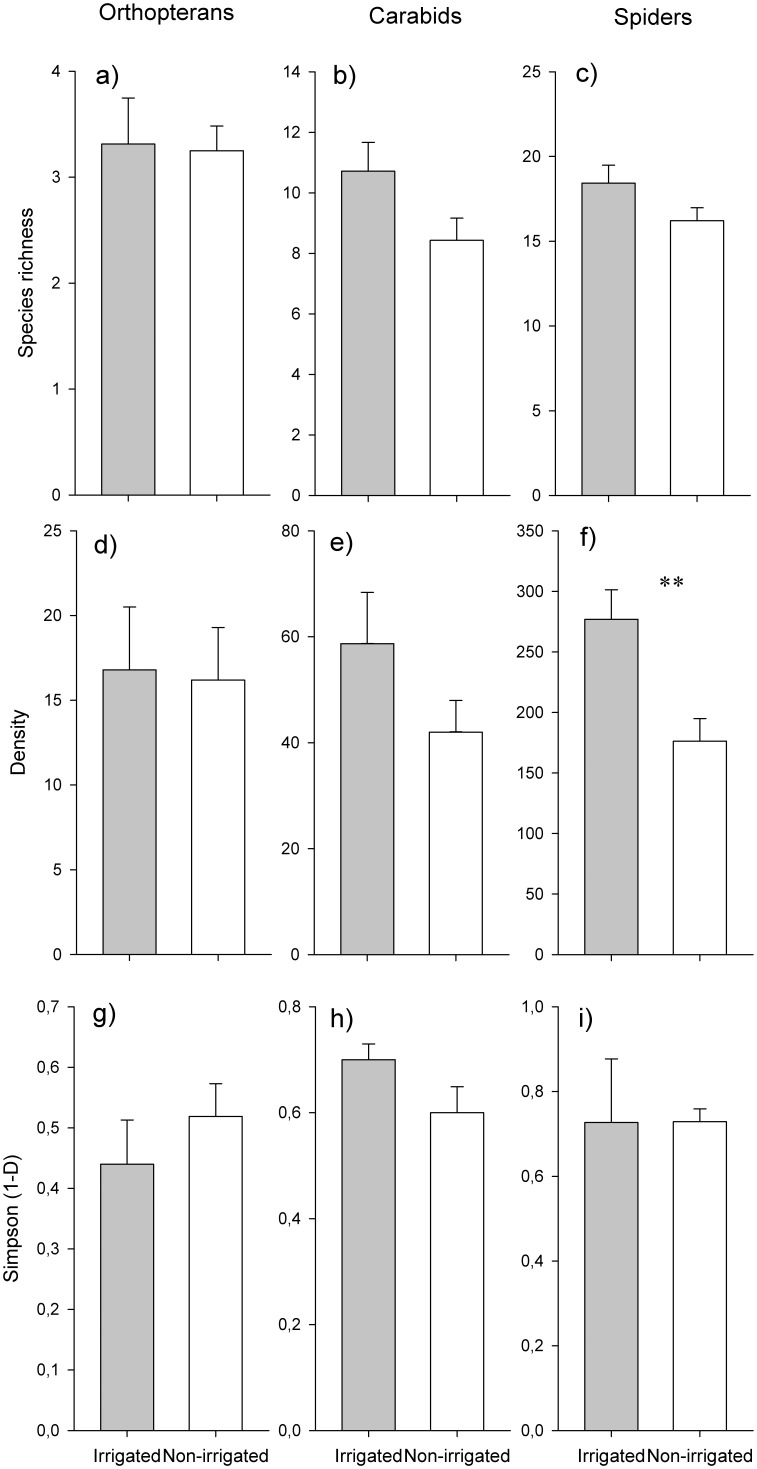
Comparison of species richness (a–c), densities (d–f), and Simpson diversity (g–i) of orthopterans, carabids, and spiders between irrigated and non-irrigated meadows (mean and SE). Differences of species richness and densities were tested with Poisson GLM’s and of Simpson diversity (1−D) with non-parametric wilcoxon rang sum tests.

A total of 7 orthopteran species (528 individuals), 47 carabid species (1,410 individuals), and 56 spider species (6,347 individuals) were found (Tables S3–S6). All 7 orthopteran species were detected in both meadow types (Fig. 2a). A total of 40 carabid species were found in irrigated meadows compared to 32 species in non-irrigated meadows (Fig. 2b). In total 49 spider species could be detected in irrigated and 46 species in non-irrigated meadows (Fig. 2c).

### Local diversity

There was no significant effect of irrigation on species richness of orthopterans (z = 0.098, P = 0.922, Fig. 2a), carabids (t = 1.950, P = 0.051; Fig. 2b), and spiders (z = 1.407, P = 0.160, Fig. 2c). While densities of orthopterans (t = 0.130, P = 0.898, Fig. 2d) and carabids (t = 1.484, P = 0.150, Fig. 2e) did not significantly differ between both meadow types densities of spiders were significantly higher in irrigated meadows (t = 3.266, P = 0.003, Fig. 2f). Similar to species richness, Simpson diversity did not differ for orthopterans (W_30_ = 147.5, P = 0.4731, Fig. 2g), carabids (W_26_ = 62, P = 0.104, Fig. 2h), and spiders (W_26_ = 87, P = 0.629, Fig. 2i).

### Community differentiation (beta diversity)

**Figure 3:**
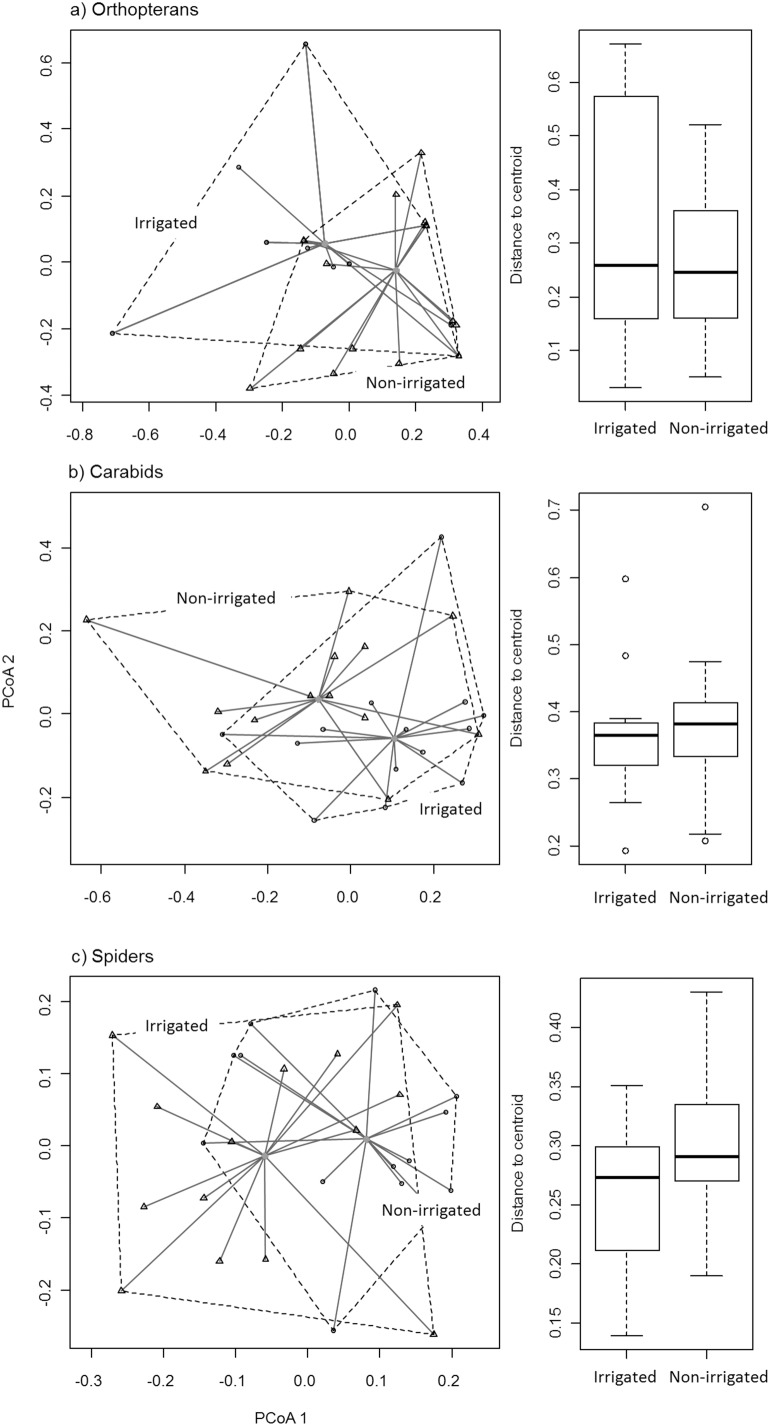
Beta diversity (multivariate homogeneity of dispersions) of a) orthopterans, b) carabid and c) spider assemblages of irrigated and non-irrigated meadows. Distances (Sørensen similarity) are reduced to principal coordinates and distances to group centroids (irrigated or non-irrigated) are shown. Differences of mean distances between meadow types were tested by ANOVA.

Beta diversity (multivariate dispersion) of all investigated taxa was not influenced by irrigation. Mean distances to centroids did not differ significantly between irrigated and non-irrigated meadows for orthopterans (F = 1.237, P = 0.275, Fig. 3a), carabids (F = 0.287, P = 0.596, Fig. 3b), and spiders (F = 4.023, P = 0.055, Fig. 3c).

### Species composition

**Figure 4:**
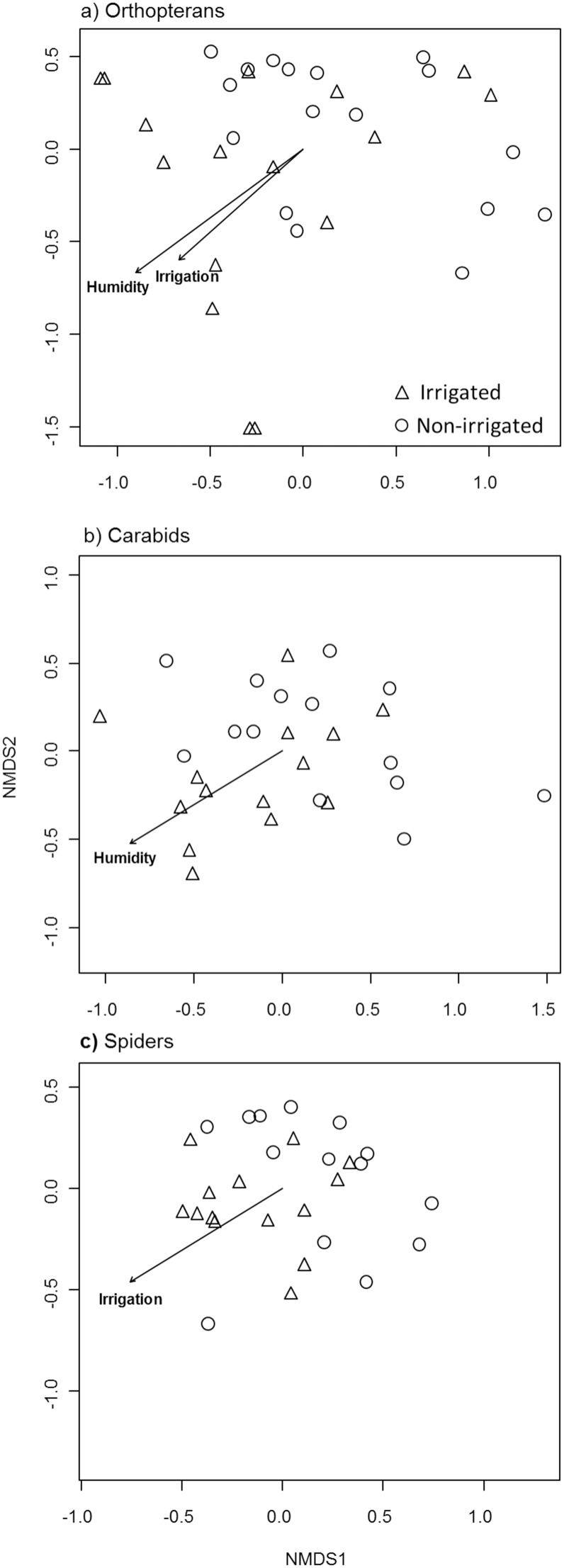
NMDS ordinations of a) orthopterans, b) carabid and c) spider species composition of irrigated and non-irrigated meadows. Only significant environmental variables are shown (permutational multivariate ANOVA, for statistics see Table 1).

Irrigation (yes or no) and humidity were the only variables having a significant effect on species composition, while fertilization, nitrogen availability, distance to permanent water and distance to forest had no effect (Table 1, Fig. 4). Orthopteran species composition was significantly affected by irrigation (F = 2.51, R^2^ = 0.073, P = 0.019) and humidity (F = 2.93, R^2^ = 0.085, P = 0.011). Carabid species composition was affected only by humidity (F = 2.49, R^2^ = 0.088, P = 0.024) while spider species composition was influenced by irrigation (F = 2.31, R^2^ = 0.080, P = 0.041).

**Table 1:** Effects of management and environmental variables on species composition of orthopterans, carabids, and spiders in irrigated and non-irrigated meadows in the Queichtal, Germany.

	Orthopterans		Carabids		Spiders	
	R^2^	P	R^2^	P	R^2^	P
**Factors**						
Irrigation (yes or no)	0.078	**0.019**	0.050	0.171	0.080	**0.041**
Fertilization (yes or no)	0.028	0.451	0.012	0.976	0.023	0.710
**Habitat parameters**						
Humidity	0.085	**0.011**	0.088	**0.024**	0.060	0.102
Nitrogen	0.023	0.568	0.029	0.582	0.033	0.451
**Landscape parameters**						
Distance to water	0.032	0.370	0.048	0.175	0.012	0.924
Distance to forest	0.031	0.405	0.027	0.613	0.061	0.121

Significance was tested by permutational multivariate ANOVA (command ‘adonis’ in R package vegan). Significant results (P<0.05) are shown in bold.

**Figure 5:**
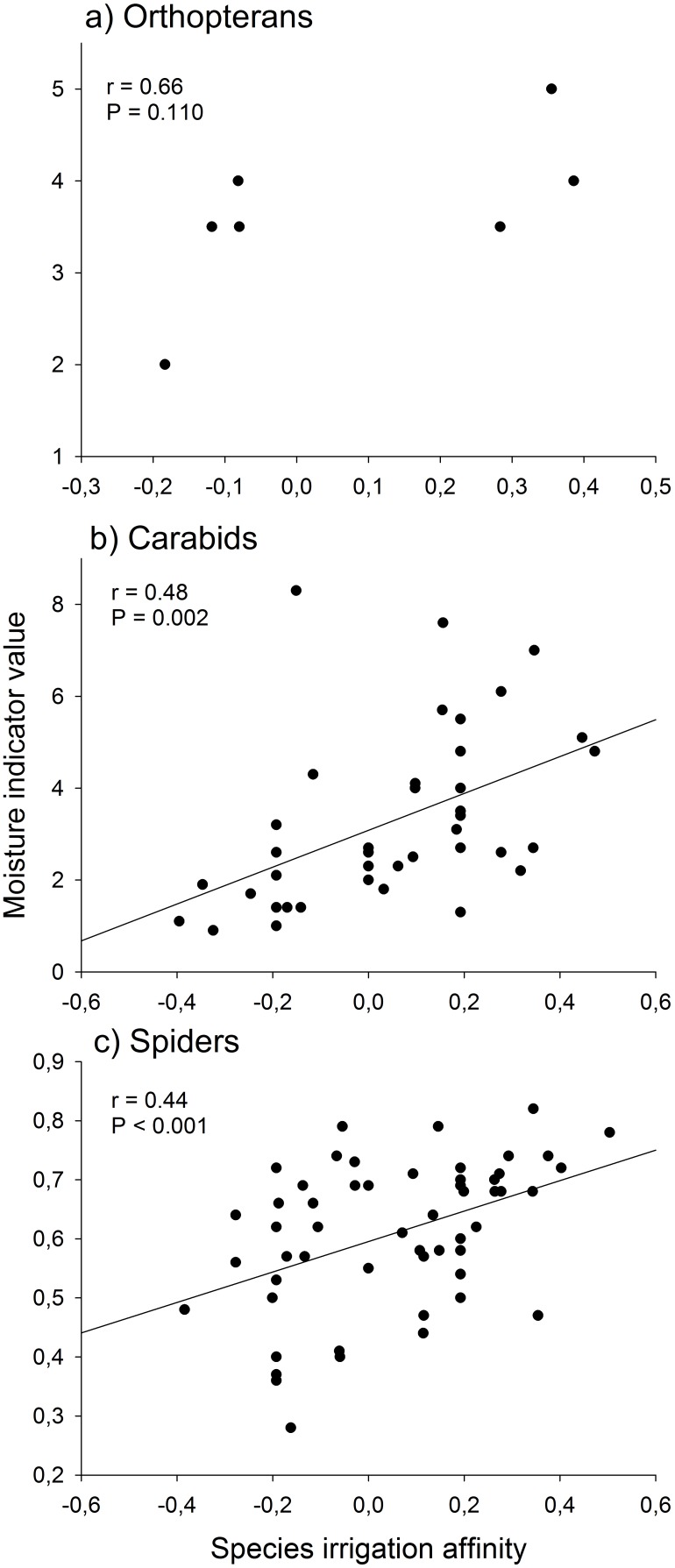
Relationship between species irrigation affinity (spearman rank correlation coefficient of species densities to irrigation) and moisture indicator value of a) orthopterans, b) carabids, and c) spiders.

As hypothesised, irrigation favoured the occurrence of moisture-dependent species. For carabids (r = 0.48, P = 0.002, Fig. 5b) and spiders (r = 0.44, P<0.001, Fig. 5c) there was a significant positive relationship between species irrigation affinity (expressed as the spearman rank correlation coefficient) and species moisture indicator value. For orthopterans no significant relationship was found, however this may be a result of the low number of N = 7 species (Fig. 5a). The combined number of species of conservation concern of all three taxa did not differ between irrigated (3.6±0.6) and non-irrigated (2.4±0.3) meadows (z = 1.853, P = 0.064).

## Discussion

Unexpectedly, traditional flood irrigation had no significant effect on diversity and species of conservation concern of orthopterans, carabids, and spiders in lowland meadows. However, flood irrigation and the associated environmental parameter humidity influenced the species composition of all taxa and shifted species assemblages towards more moisture-dependent species.

### Local diversity

We assumed that traditional flood irrigation leads to small-scale differences in moisture and sediment conditions and consequently higher microhabitat and vegetation heterogeneity resulting in increased local diversity [16], [48]. However, in contrast to our hypothesis, traditionally flood irrigated meadows did not contain a higher local diversity (in terms of species richness and Simpson diversity) of orthopterans, carabids, and spiders compared to non-irrigated meadows. Possibly, the traditional irrigation system in our study area - on average four flooding events with a maximum of twelve irrigation days between April and August - is not sufficient to induce (measurable) heterogeneity effects. Riedener et al. [16] assumed that effects of the irrigation technique on diversity are only effective in combination with other management factors such as mowing and grazing regimes. Additionally, landscape variables such as patch isolation can influence species [32]. We accounted for possible confounding effects of management (mowing frequency, fertilization) and landscape parameters (distance to permanent water, distance to forest), none of which differed significantly between irrigated and non-irrigated meadows. However, as in any observational study, we cannot rule out that additional unmeasured management or environmental parameters have influenced our results.

In the literature, several studies in riparian habitats were able to detect positive effects of (natural) flooding on diversity [48]–[51]. However, in contrast to our study system with no (non-irrigated) and low intensive (irrigated) flooding, flood intensities in these studies are mostly studied along gradients containing higher intensities. Gerisch et al. [50] found positive effects for carabids at the river Elbe (Germany) and explained this by higher resource diversity in frequently flooded habitats. At the river Meuse (Belgium/Netherlands), Lambeets [50] could show that flooding initially had a positive effect on carabid diversity, which peaked at intermediate flooding degrees. This is in line with findings of Pollack et al. [48] in riparian meadows where plant species richness was highest at intermediately flooded river banks because of increased microhabitat heterogeneity.

Similar to diversity, densities of orthopterans and carabids did not differ between meadow types. However, spider densities were higher in irrigated meadows. One explanation might be enhanced food availability, because short time flooding can enhance soil organisms [52], which present important food source especially for linyphiid spiders [53].

### Community differentiation (beta diversity)

Irrigation did not influence community differentiation and, in contrast to our hypothesis, beta diversity of orthopterans, carabids, and spiders was not higher in irrigated compared to non-irrigated meadows. Although flood irrigation between irrigated meadows differed in time and intensity, these differences were obviously too weak to result in more diverse species assemblages. Moreover, the traditional flooding method in the region - where the dammed river water slowly streams into the meadows and back into the river through a system of open ditches - leads to a relatively homogenous water flow. This prevents stagnant moisture [12] and moisture conditions on irrigated sites might be more uniform than expected. Human-altered repetitive flood events are known to result in uniform species compositions due to a homogenization of habitat structure [54], [55].

### Species composition

As hypothesized, meadow irrigation and the associated altered humidity conditions influenced species composition of orthopterans, carabids, and spiders. Irrigation may therefore increase beta diversity at the landscape scale and contribute to diverse grassland communities. Assemblages of irrigated meadows contained more moisture-dependent species compared to non-irrigated ones. This was reflected for carabids and spiders by the positive relationships between species irrigation affinity and their moisture indicator value. For orthopterans this effect was not significant (most likely because of the low number of species), but the two species with the highest moisture indicator value – *Mecostethus parapleurus* and *Stetophyma grossum* – were significantly more abundant in irrigated meadows (Table S3). For all three taxa, humidity is known to be one of the most influencing environmental parameter structuring species compositions [27], [43], [56]. Impacts of (natural) flood disturbance on species and trait composition of orthopterans were previously shown by Dziock et al. [57]. Bonn et al. [48] found that flood regime strongly influenced carabid species assemblages, which was also found by Lambeets et al. [50] for carabid and spider communities. Although fertilization can strongly influence arthropods [58], [59] we could not detect an effect of fertilization. In general, fertilization application rates were low in the study area (0 to ∼60 kg N•ha-1•yr-1), a range in which also plants showed no significant decrease in species richness (unpublished data). Similar to fertilization, the landscape parameters, distance to forest and to permanent water, did not differ between irrigated and non-irrigated meadows and had no effect on species compositions, respectively.

In contrast to our hypothesis, species compositions of flood irrigated meadows did not contain more species of conservation concern than non-irrigated meadows. This is in contrast to Bonn et al. [49] and Lambeets et al. [51], where anthropogenic alterations in flooding regimes not only have a strong influence on arthropod communities but also on the distribution of rare (and often endangered) riparian species. Again, this difference may be due to the low irrigation intensity in our study system.

### Conclusion

Flood irrigation had no significant effect on local and beta diversity of orthopterans, carabids, and spiders in lowland meadows. In contrast, flood irrigation clearly changed species assemblages towards moisture-dependent species and probably increased beta diversity at the landscape scale. However, these species were mostly common species and assemblages of irrigated meadows did not contain more species of conservation concern compared to non-irrigated ones. More variable and intensive (higher duration and/or frequency) flooding regimes are likely to provide much stronger conservation benefits. Moreover, beneficial effects of flood irrigation might be more pronounce along the irrigation infrastructures (open ditches, drains, weirs) than in the open meadow, which will be tested in further studies.

## Supporting Information

Table S1 **Site characteristics of irrigated and non-irrigated meadows in the ‘Queichtal’, Germany.** For explanations see text.
(DOCX)

Table S2 **Moisture indicator values of orthopterans based on information in Maas et al. (2002).** Information was coded numerically as follows: Strongly xerophilic = 1, xerophilic = 2, mesophilic = 3, hygrophilic = 4, strongly hygrophilic = 5.
(DOCX)

Table S3 **Abundances (mean and SE) of orthopterans, carabid, and spider species in irrigated and non-irrigated meadows in the Queichtal, Rhineland-Palatine (Germany).** Differences were tested with Poisson or, in case of overdispersion, quasi-Poisson GLM’s for count data (only species with ≥10 individuals). n.t. = not tested. Significant results (P<0.05) are shown in bold.
(DOCX)

Table S4 **Additional data.**
(XLSX)

Table S5 **Additional data.**
(XLSX)

Table S6 **Additional data.**
(XLSX)
