# Supplementary material for: Effects of Traditional Flood Irrigation on Invertebrates in Lowland Meadows
Source: PLoS One. 2014 Oct 23;9(10):e110854. doi: 10.1371/journal.pone.0110854 (PMC4207796; doi:10.1371/journal.pone.0110854)
Supplement: Table S1 — Site characteristics of irrigated and non-irrigated meadows in the ‘Queichtal’, Germany. For explanations see text. (DOCX) [file pone.0110854.s001.docx]

Table S1: Site characteristics of irrigated and non-irrigated meadows in the ‘Queichtal’, Germany. For explanations see text.

| **Plot Id** | **X** | **Y** | **Irrigation** | **Fertilization** | **Distance to**  **permanent water [m]** | **Distance to**  **forest [m]** | **Humidity** | **Nitrogen** |
| --- | --- | --- | --- | --- | --- | --- | --- | --- |
| 1-01-a | 440119,65 | 5450296,47 | yes | yes | 203 | 130 | 5,83 | 5,40 |
| 1-02-a | 442300,00 | 5450782,00 | yes | yes | 88 | 61 | 5,69 | 5,61 |
| 1-03-a | 442974,37 | 5450377,51 | yes | yes | 684 | 84 | 5,83 | 5,05 |
| 1-04-a | 442883,60 | 5450863,09 | yes | yes | 215 | 137 | 5,45 | 5,29 |
| 1-08-a | 444934,00 | 5450805,00 | yes | yes | 80 | 71 | 5,98 | 5,63 |
| 1-09-a | 445282,91 | 5451473,93 | yes | yes | 160 | 55 | 5,66 | 5,35 |
| 2-02-a | 446395,00 | 5450898,00 | yes | yes | 716 | 165 | 5,70 | 5,27 |
| 3-02-a | 440521,38 | 5450779,82 | yes | yes | 590 | 68 | 5,86 | 5,80 |
| 3-04-a | 446822,59 | 5450857,02 | yes | yes | 1100 | 140 | 5,64 | 5,33 |
| 1-04-b | 443024,75 | 5450613,10 | yes | no | 450 | 95 | 5,59 | 5,89 |
| 1-05-a | 443803,00 | 5450380,42 | yes | no | 737 | 56 | 5,86 | 5,09 |
| 1-05-b | 444742,00 | 5450338,00 | yes | no | 407 | 40 | 5,52 | 5,64 |
| 1-06-a | 444858,48 | 5450193,87 | yes | no | 494 | 55 | 5,34 | 5,82 |
| 1-07-b | 444628,04 | 5451087,24 | yes | no | 97 | 93 | 6,22 | 5,84 |
| 3-03-a | 444173,62 | 5451238,30 | yes | no | 187 | 182 | 6,17 | 4,91 |
| 3-04-c | 446909,00 | 5450993,00 | yes | no | 1230 | 265 | 5,94 | 5,87 |
| 5-05-b | 442297,67 | 5451085,00 | no | yes | 157 | 160 | 5,84 | 5,80 |
| 5-05-c | 443130,82 | 5451403,30 | no | yes | 190 | 38 | 5,61 | 5,14 |
| 5-06-a | 443214,78 | 5450628,43 | no | yes | 488 | 55 | 5,48 | 5,23 |
| 5-07-a | 447181,62 | 5452342,49 | no | yes | 58 | 56 | 5,42 | 5,98 |
| 5-07-b | 447750,00 | 5452204,00 | no | yes | 188 | 55 | 5,32 | 5,21 |
| 5-11-a | 445472,00 | 5449688,00 | no | yes | 583 | 233 | 5,76 | 4,86 |
| 5-11-b | 445184,00 | 5450037,00 | no | yes | 675 | 75 | 5,49 | 5,07 |
| 5-13-b | 446880,00 | 5451195,00 | no | yes | 1285 | 136 | 5,69 | 5,61 |
| 5-03-a | 439372,42 | 5450103,82 | no | no | 63 | 42 | 5,85 | 5,17 |
| 5-05-a | 443851,34 | 5451497,59 | no | no | 167 | 231 | 5,57 | 5,35 |
| 5-08-a | 443979,73 | 5452477,74 | no | no | 85 | 47 | 5,97 | 5,96 |
| 5-08-b | 444286,98 | 5452617,65 | no | no | 198 | 75 | 5,62 | 5,00 |
| 5-09-a | 444466,20 | 5451955,01 | no | no | 545 | 144 | 5,69 | 5,09 |
| 5-10-a | 444812,00 | 5451322,00 | no | no | 100 | 56 | 5,94 | 5,52 |
| 5-12-a | 446013,00 | 5450841,00 | no | no | 350 | 39 | 5,76 | 5,42 |
| 5-13-a | 447913,82 | 5451089,43 | no | no | 1195 | 53 | 5,26 | 5,59 |
