# Supplementary material for: Effects of Traditional Flood Irrigation on Invertebrates in Lowland Meadows
Source: PLoS One. 2014 Oct 23;9(10):e110854. doi: 10.1371/journal.pone.0110854 (PMC4207796; doi:10.1371/journal.pone.0110854)
Supplement: Table S2 — Moisture indicator values of orthopterans based on information in Maas et al. (2002). Information was coded numerically as follows: Strongly xerophilic = 1, xerophilic = 2, mesophilic = 3, hygrophilic = 4, strongly hygrophilic = 5. (DOCX) [file pone.0110854.s002.docx]

Table S2: Moisture indicator values of orthopterans based on information in Maas et al. (2002). Information was coded numerically as follows: Strongly xerophilic = 1, xerophilic = 2, mesophilic = 3, hygrophilic = 4, strongly hygrophilic = 5.

| **Species** | **Maas et al. (2002)** | **Moisture indicator value** |
| --- | --- | --- |
| *Aiolopus thalassinus* | Hygrophilic | 4 |
| *Chorthippus biguttulus* | Xerophilic | 2 |
| *Chorthippus dorsatus* | Mesophilic-hygrophilic | 3.5 |
| *Chorthippus parallelus* | Mesophilic-hygrophilic | 3.5 |
| *Mecostethus parapleurus* | Hygrophilic | 4 |
| *Metrioptera roeselii* | Mesophilic-hygrophilic | 3.5 |
| *Stetophyma grossum* | Strongly hygrophilic | 5 |
