# Supplementary material for: Effects of Traditional Flood Irrigation on Invertebrates in Lowland Meadows
Source: PLoS One. 2014 Oct 23;9(10):e110854. doi: 10.1371/journal.pone.0110854 (PMC4207796; doi:10.1371/journal.pone.0110854)
Supplement: Table S3 — Abundances (mean and SE) of orthopterans, carabid, and spider species in irrigated and non-irrigated meadows in the Queichtal, Rhineland-Palatine (Germany). Differences were tested with Poisson or, in case of overdispersion, quasi-Poisson GLM’s for count data (only species with ≥10 individuals). n.t. = not tested. Significant results (P<0.05) are shown in bold. (DOCX) [file pone.0110854.s003.docx]

Table S3: Abundances (mean and SE) of orthopterans, carabid, and spider species in irrigated and non-irrigated meadows in the Queichtal, Rhineland-Palatine (Germany). Differences were tested with Poisson or, in case of overdispersion, quasi-Poisson GLM’s for count data (only species with ≥ 10 individuals). n.t. = not tested. Significant results (P < 0.05) are shown in bold.

| **Species** | **Abundance** | | **Statistics** | |
| --- | --- | --- | --- | --- |
|  | **Irrigated** | **Non-irrigated** | **t/z** | **P** |
| Orthopterans |  |  |  |  |
| *Aiolopus thalassinus* | 0.1 ± 0.1 | 0.1 ± 0.1 | n.t. |  |
| *Chorthippus biguttulus* | 2.6 ± 1.0 | 4.8 ± 1.9 | t = -1.051 | 0.302 |
| *Chorthippus dorsatus* | 5.9 ± 2.6 | 7.3 ± 1.8 | t = -0.428 | 0.672 |
| *Chorthippus parallelus* | 1.3 ± 0.4 | 1.8 ± 0.5 | t = -0.653 | 0.519 |
| *Mecostethus parapleurus* | 2.3 ± 0.8 | 0.4 ± 0.1 | t = 2.492 | **0.019** |
| *Metrioptera roeselii* | 0.6 ± 0.3 | 0.1 ± 0.1 | z = 2.084 | **0.037** |
| *Stetophyma grossum* | 4.2 ± 1.0 | 1.8 ± 0.5 | t = 2.091 | **0.045** |
|  |  |  |  |  |
| Carabids |  |  |  |  |
| *Abax parallelepipedus* | 0.1 ± 0.1 | 0 ± 0 | n.t. |  |
| *Agonum muelleri* | 0.1 ± 0.1 | 0 ± 0 | n.t. |  |
| *Agonum viduum* | 0.1 ± 0.1 | 0 ± 0 | n.t. |  |
| *Amara aenea* | 1.4 ± 0.7 | 3.6 ± 1.5 | t = -1.313 | 0.200 |
| *Amara communis* | 1.1 ± 0.4 | 0.9 ± 0.4 | t = 0.486 | 0.631 |
| *Amara familiaris* | 0.1 ± 0.1 | 0.1 ± 0.1 | n.t. |  |
| *Amara kultii* | 0.8 ± 0.3 | 0.7 ± 0.4 | t = 0.136 | 0.893 |
| *Amara lunicollis* | 4.0 ± 0.9 | 3.1 ± 0.7 | t = 0.806 | 0.428 |
| *Amara plebeja* | 0.1 ± 0.1 | 0 ± 0 | n.t. |  |
| *Amara similata* | 0.1 ± 0.1 | 0.1 ± 0.1 | n.t. |  |
| *Amara strenuua* | 0.4 ± 0.3 | 0 ± 0 | n.t. |  |
| *Amara tibialis* | 0 ± 0 | 0.1 ± 0.1 | n.t. |  |
| *Anisodactylus binotatus* | 3.1 ± 0.9 | 2.3 ± 1.5 | t = 0.475 | 0.639 |
| *Bembidion guttula* | 2.4 ± 0.9 | 0.2 ± 0.2 | t = 2.369 | **0.026** |
| *Bembidion lampros* | 0.4 ± 0.3 | 0 ± 0 | n.t. |  |
| *Bembidion properans* | 0.1 ± 0.1 | 0.1 ± 0.1 | n.t. |  |
| *Bembidion quadrimaculatum* | 0 ± 0 | 0.1 ± 0.1 | n.t. |  |
| *Brachinus explodens* | 0.1 ± 0.1 | 0 ± 0 | n.t. |  |
| *Carabus cancellatus* | 0.5 ± 0.3 | 0.4 ± 0.4 | t = 0.160 | 0.874 |
| *Carabus granulatus* | 0.9 ± 0.3 | 0.1 ± 0.1 | t = 2.255 | **0.033** |
| *Carabus nemoralis* | 0 ± 0 | 0.1 ± 0.1 | n.t. |  |
| *Carabus violaceus* | 0.1 ± 0.1 | 0 ± 0 | n.t. |  |
| *Chlaenius nigricornis* | 0.3 ± 0.2 | 0 ± 0 | n.t. |  |
| *Clivina collaris* | 0.1 ± 0.1 | 0.1 ± 0.1 | n.t. |  |
| *Clivina fossor* | 0.6 ± 0.2 | 0.3 ± 0.2 | t = 0.870 | 0.392 |
| *Diachromus germanus* | 0.1 ± 0.1 | 0 ± 0 | n.t. |  |
| *Dyschirius globosus* | 0.4 ± 0.3 | 0.1 ± 0.1 | n.t. |  |
| *Harpalus affinis* | 0.4 ± 0.1 | 0.4 ± 0.2 | z = 0.000 | 1.000 |
| *Harpalus anxius* | 0.3 ± 0.2 | 0 ± 0 | n.t. |  |
| *Harpalus distinguendes* | 0.3 ± 0.1 | 0.1 ± 0.1 | n.t. |  |
| *Harpalus latus* | 0.2 ± 0.2 | 0.4 ± 0.2 | n.t. |  |
| *Harpalus luteicornis* | 0.2 ± 0.2 | 0.9 ± 0.3 | t = -1.935 | 0.064 |
| *Harpalus rubripes* | 0 ± 0 | 0.1 ± 0.1 | n.t. |  |
| *Loricera pililcornis* | 0.1 ± 0.1 | 0 ± 0 | n.t. |  |
| *Microlestes minutulus* | 0.2 ± 0.2 | 0 ± 0 | n.t. |  |
| *Oodes helopioides* | 0.4 ± 0.3 | 0 ± 0 | n.t. |  |
| *Ophonus ardosiacus* | 0 ± 0 | 0.1 ± 0.1 | n.t. |  |
| *Poecilus cupreus* | 11.1 ± 4.4 | 2.6 ± 1.2 | t = 1.985 | 0.058 |
| *Poecilus versicolor* | 25.9 ± 6.8 | 21.7 ± 5.4 | t = 0.479 | 0.636 |
| *Pseudophonus rufipes* | 0 ± 0 | 0.2 ± 0.1 | n.t. |  |
| *Pterostichus anthracinus* | 0.1 ± 0.1 | 0 ± 0 | n.t. |  |
| *Pterostichus diligens* | 0.2 ± 0.1 | 0.4 ± 0.3 | n.t. |  |
| *Pterostichus melanarius* | 1.9 ± 0.7 | 1.6 ± 0.6 | t = 0.318 | 0.753 |
| *Pterostichus strenuus* | 0.1 ± 0.1 | 0 ± 0 | n.t. |  |
| *Pterostichus vernalis* | 0.1 ± 0.1 | 0.1 ± 0.1 | n.t. |  |
| *Stomis pumicatus* | 0 ± 0 | 0.1 ± 0.1 | n.t. |  |
| *Syntomus truncatellus* | 0.4 ± 0.2 | 0.7 ± 0.3 | t = -0.870 | 0.392 |
|  |  |  |  |  |
| Spiders |  |  |  |  |
| *Agyneta affinis* | 1.1 ± 0.4 | 1.9 ± 0.7 | t = -0.997 | 0.328 |
| *Agyneta rurestris* | 1.0 ± 0.3 | 1.4 ± 0.6 | t = -0.554 | 0.584 |
| *Alopecosa cuneata* | 20.9 ± 6.7 | 23.4 ± 5.0 | t = -0.307 | 0.761 |
| *Alopecosa pulverulenta* | 0.1 ± 0.1 | 0 ± 0 | n.t. |  |
| *Araeoncus humilis* | 0.1 ± 0.1 | 0 ± 0 | n.t. |  |
| *Arctosa leopardus* | 8.5 ± 3.6 | 0.4 ± 0.3 | t = 2.094 | **0.046** |
| *Arctosa lutetiana* | 0.1 ± 0.1 | 0.1 ± 0.1 | n.t. |  |
| *Arctosa perita* | 0.1 ± 0.1 | 0 ± 0 | n.t. |  |
| *Aulonia albimana* | 0 ± 0 | 0.4 ± 0.2 | n.t. |  |
| *Bathyphantes gracilis* | 0 ± 0 | 0.1 ± 0.1 | n.t. |  |
| *Ceratinella brevipes* | 0.6 ± 0.4 | 0.7 ± 0.3 | t = -0.268 | 0.791 |
| *Cnephalocotes obscurus* | 0.1 ± 0.1 | 0 ± 0 | n.t. |  |
| *Dendryphantes rudis* | 0 ± 0 | 0.1 ± 0.1 | n.t. |  |
| *Dicymbium nigrum* | 0.1 ± 0.1 | 0.1 ± 0.1 | n.t. |  |
| *Diplostyla concolor* | 0.3 ± 0.2 | 0.1 ± 0.1 | n.t. |  |
| *Drassyllus lutetianus* | 0.2 ± 0.1 | 0.1 ± 0.1 | n.t. |  |
| *Drassyllus praeficus* | 0.1 ± 0.1 | 0.2 ± 0.2 | n.t. |  |
| *Drassyllus pusillus* | 0.4 ± 0.2 | 1.1 ± 0.6 | t = -1.112 | 0.276 |
| *Erigone atra* | 2.4 ± 0.8 | 1.0 ± 0.5 | t = 1.397 | 0.174 |
| *Erigone dentipalpis* | 2.3 ± 0.6 | 4.1 ± 2.6 | t = -0.772 | 0.447 |
| *Hahnia nava* | 1.2 ± 0.5 | 0.8 ± 0.5 | t = 0.570 | 0.574 |
| *Haplodrassus signifer* | 0 ± 0 | 0.1 ± 0.1 | n.t. |  |
| *Mermessus trilobatus* | 0.2 ± 0.1 | 1.7 ± 1.0 | t = -1.646 | 0.112 |
| *Micaria pulicaria* | 0 ± 0 | 0.1 ± 0.1 | n.t. |  |
| *Oedothorax fuscus* | 31.5 ± 9.5 | 3.0 ± 1.3 | t = 2.968 | **0.006** |
| *Oedothorax retusus* | 6.9 ± 3.0 | 1.1 ± 0.1 | t = 1.913 | 0.067 |
| *Ozyptila simplex* | 2.9 ± 0.9 | 2.4 ± 1.1 | t = 0.356 | 0.725 |
| *Pachygnatha clercki* | 1.8 ± 0.8 | 0.1 ± 0.1 | t = 1.77 | 0.088 |
| *Pachygnatha degeeri* | 15.9 ± 3.1 | 11.1 ± 2.7 | t = 1.168 | 0.253 |
| *Pardosa cf agrestis* | 0.5 ± 0.4 | 0 ± 0 | n.t. |  |
| *Pardosa amentata* | 0.3 ± 0.2 | 0.4 ± 0.1 | n.t. |  |
| *Pardosa hortensis* | 0 ± 0 | 0.1 ± 0.1 | n.t. |  |
| *Pardosa lugubris* | 0.1 ± 0.1 | 0 ± 0 | n.t. |  |
| *Pardosa palustris* | 109.0 ± 20.1 | 70.7 ± 17.9 | t = 1.375 | 0.181 |
| *Pardosa prativaga* | 8.1 ± 2.5 | 3.4 ± 1.7 | t = 1.505 | 0.144 |
| *Pardosa pullata* | 30.3 ± 6.6 | 24.2 ± 5.8 | t = 0.694 | 0.494 |
| *Pelecopsis parallela* | 2.8 ± 1.0 | 3.1 ± 1.7 | t = -0.145 | 0.886 |
| *Phrurolithus festivus* | 0.1 ± 0.1 | 0.1 ± 0.1 | n.t. |  |
| *Phrurolithus minimus* | 0 ± 0 | 0.1 ± 0.1 | n.t. |  |
| *Pirata latitans* | 0.1 ± 0.1 | 0 ± 0 | n.t. |  |
| *Pirata piraticus* | 0.3 ± 0.2 | 0.1 ± 0.1 | n.t. |  |
| *Pirata uliginosus* | 0.4 ± 0.2 | 0.5 ± 0.4 | t = -0.150 | 0.882 |
| *Tenuiphantes tenius* | 0.1 ± 0.1 | 0 ± 0 | n.t. |  |
| *Thanatus striatus* | 0 ± 0 | 0.1 ± 0.1 | n.t. |  |
| *Tiso vagans* | 0.1 ± 0.1 | 0.1 ± 0.1 | n.t. |  |
| *Trachyzelotes pedestris* | 0.1 ± 0.1 | 0.2 ± 0.2 | n.t. |  |
| *Trochosa ruricola* | 21.1 ± 3.7 | 12.8 ± 2.6 | t = 1.869 | 0.073 |
| *Trochosa spinipalpis* | 0.2 ± 0.2 | 0 ± 0 | n.t. |  |
| *Trochosa terricola* | 0.4 ± 0.2 | 0.8 ± 0.4 | t = -0.900 | 0.376 |
| *Walckenaeria antica* | 0 ± 0 | 0.1 ± 0.1 | n.t. |  |
| *Xerolycosa miniata* | 1.4 ± 0.6 | 2.3 ± 1.2 | t = -0.708 | 0.485 |
| *Xysticus acerbus* | 1.6 ± 0.4 | 0.7 ± 0.3 | t = 1.874 | 0.072 |
| *Xysticus audax* | 0.3 ± 0.1 | 0.3 ± 0.2 | n.t. |  |
| *Xysticus cristatus* | 0.5 ± 0.2 | 0.3 ± 0.2 | t = 0.702 | 0.489 |
| *Xysticus kochi* | 0.4 ± 0.2 | 0.3 ± 0.2 | t = 0.547 | 0.589 |
| *Zelotes latreillei* | 0.1 ± 0.1 | 0 ± 0 | n.t. |  |
